# Distal Clavicle Plate Combined With EndoButton Device for the Treatment of Neer Type IIB Distal Clavicle Fractures

**DOI:** 10.1002/atn2.70379

**Published:** 2026-11-03

**Authors:** Tianhao Zhu, Changming Huang, Huaqiang Fan, Xichun Hu, Yangpan Fu, Huixiang Dong, Yaqing Zhang

**Affiliations:** ^1^ Department of Orthopedics Hospital of PLA 73rd Group Army, Chenggong Hospital Affiliated to Xiamen University Xiamen China

## Abstract

The present article describes a surgical technique for the management of Neer type IIB distal clavicle fractures using a distal clavicle plate combined with an EndoButton device. The procedure consists of stepwise arthroscopic evaluation and exposure, fracture reduction, conoid ligament reconstruction, and plate fixation. This combined approach allows for concurrent management of intra‐articular lesions, provides stable fixation, reduces the risk of iatrogenic clavicle fractures, and facilitates early postoperative rehabilitation. Nevertheless, it has certain limitations: the approach is not minimally invasive, its indications are relatively narrow, and it requires advanced arthroscopic expertise. All in all, this surgical approach is applicable to both acute and chronic Neer type IIB distal clavicle fractures.

## Graphical abstract

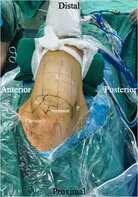

**VIDEO 1:** The detailed procedure of surgery. The operation was performed on the right shoulder with the patient in the left lateral position. Video content can be viewed at https://doi.org/10.1002/atn2.70379.

Distal clavicle fractures (DCFs) are relatively uncommon, accounting for approximately 10% to 30% of all clavicle fractures.^1^ Neer initially classified clavicle fractures into 5 types, and Craig et al. subsequently subdivided type II into type IIA and type IIB, with the latter being characterized by disruption of the conoid ligament.^1^ Owing to the typically small distal fragment in Neer type IIB fractures, the optimal fixation strategy remains controversial. The reported nonunion rate after conservative treatment ranges from about 22% to 50%, and around 14% of patients remain symptomatic.^2^ Various surgical approaches have been described, including Kirschner wire fixation, coracoclavicular screw fixation, hook plate fixation, distal clavicle plate fixation, and suspensory techniques. Nonetheless, the use of any single modality alone is often associated with complications such as fixation failure, nonunion, and subacromial impingement syndrome, or the need for secondary surgery for implant removal.^3^ In recent years, increasing evidence has supported the combined use of a distal clavicle plate with a coracoclavicular suspensory technique for Neer type IIB DCFs, showing superior biomechanical stability, lower complication rates, and improved functional outcomes.^4^, ^5^ Herein, we describe a modified surgical technique for the treatment of Neer type IIB DCFs using an EndoButton device in combination with a distal clavicle plate, with the aim of providing a reference for clinical application.

## SURGICAL TECHNIQUE

### Preoperative Preparation

After satisfactory anesthesia is achieved, the patient is positioned in the lateral decubitus position. The relevant surgical landmarks are identified and marked. After skin preparation and sterile draping, the affected limb is abducted to 45°, and traction is applied using an appropriate weight (Figure 1).

**FIGURE 1:**
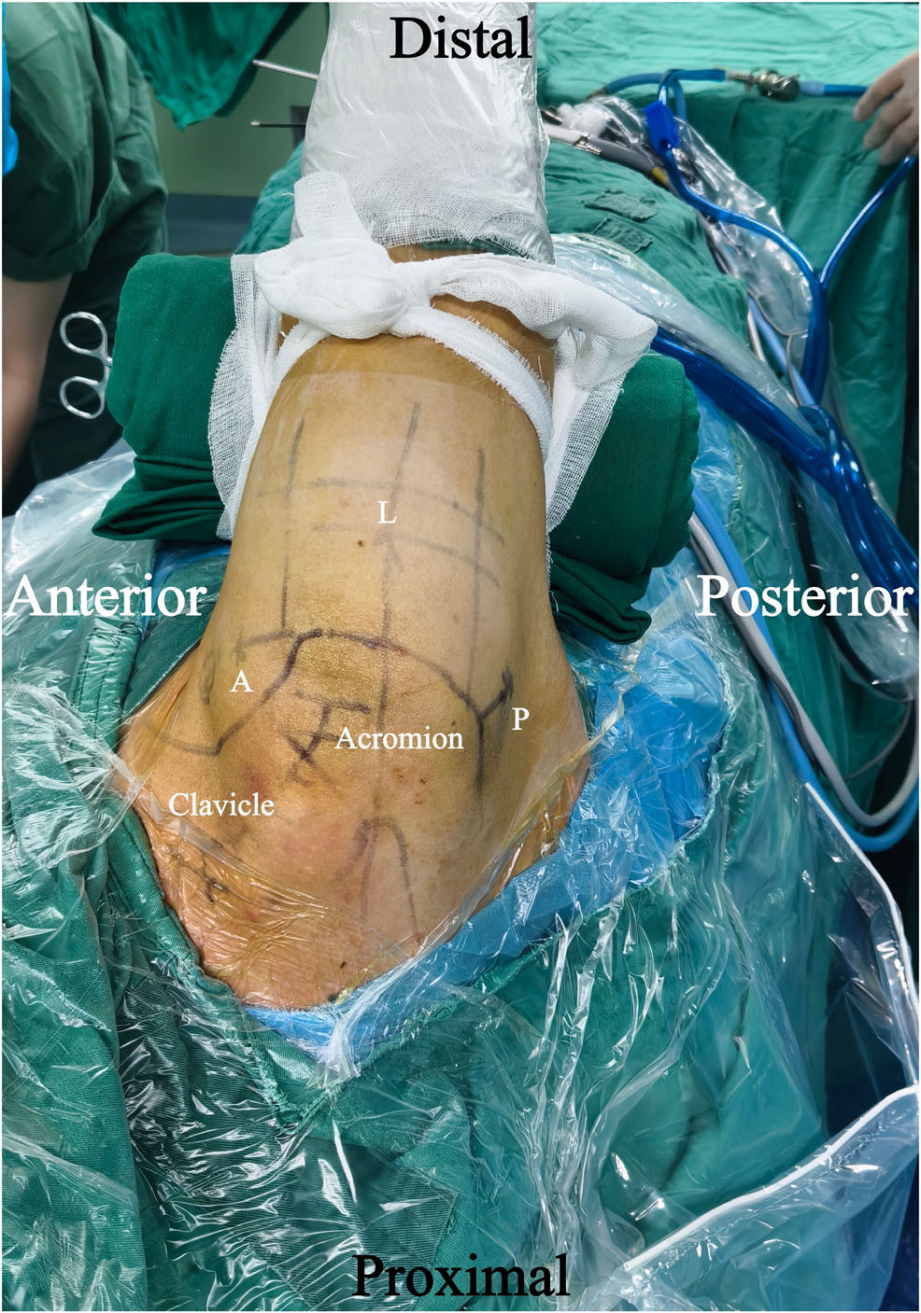
Preoperative marking (right shoulder, left lateral position). (A, anterior portal; L, lateral portal; P, posterior portal.)

### Arthroscopic Exploration

The affected shoulder is accessed through the anterior, lateral, and posterior portals, which serve as working portals. Intra‐articular exploration is performed with a 30° arthroscope (Smith & Nephew, Andover, MA) to expose the coracoid process and address any concomitant intra‐articular lesions (Video 1).

### Reduction of the Fracture

Next, an incision of approximately 6 to 7 cm is made along the distal clavicle to expose the fracture site gradually. A nonabsorbable surgical suture (Delta Medical, Beijing, China) is then used to create a Nice knot for temporary fixation (Figures 2 and 3).

**FIGURE 2:**
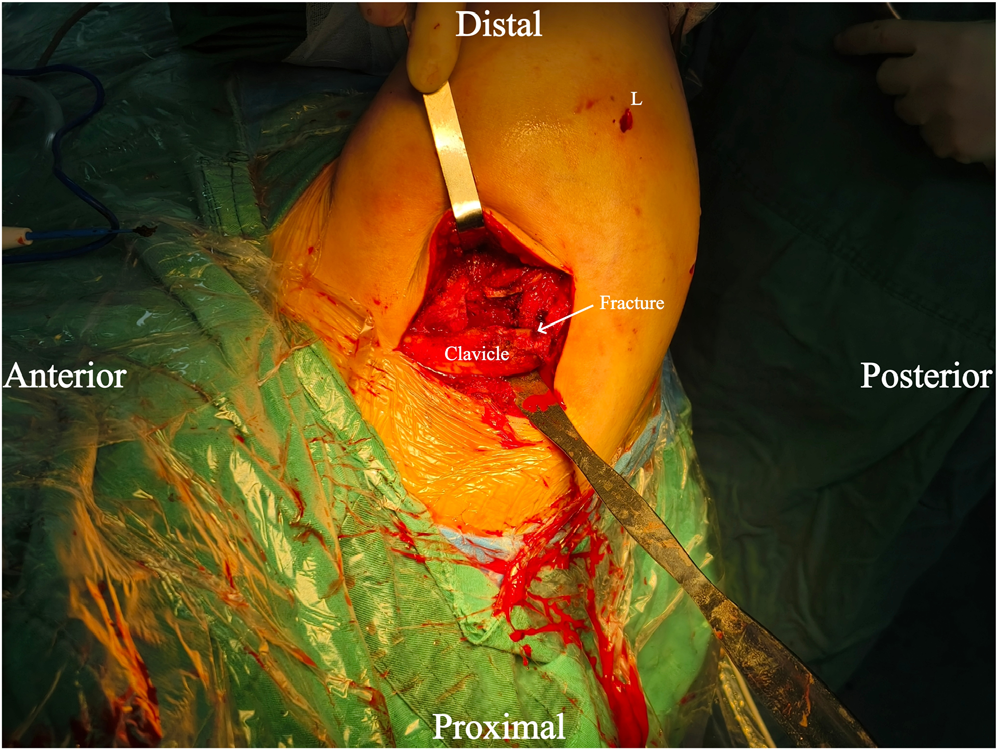
Exposure of the fracture (right shoulder, left lateral position). (L, lateral portal.)

**FIGURE 3:**
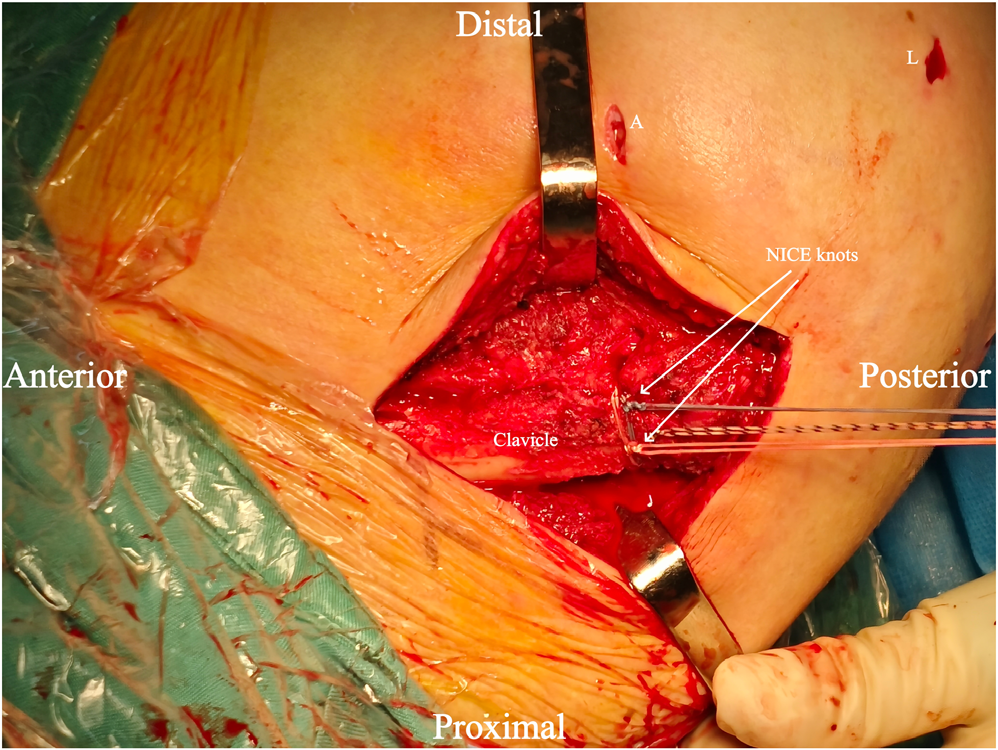
Fracture fixation with a Nice knot (right shoulder, left lateral position). (A, anterior portal; L, lateral portal.)

### Conoid Ligament Tunnel Preparation

A custom‐made temporary guide is placed at the acromioclavicular joint and secured with a screw. A 2‐mm Kirschner wire is drilled through the clavicle and coracoid process at a distance of approximately 3 to 4 cm from the distal clavicle, as marked on the guide. The tunnel is subsequently reamed to 4.5 mm, and the drill bit is retained within the tunnel. A retraction suture (Covidien, Mansfield, MA) is then passed through the hollow drill bit, and the EndoButton device (Smith & Nephew) is introduced through the tunnel with the loop positioned at the clavicular end (Figures 4 and 5). If the coracoid tunnel is not centered, the traction suture is retrieved in the reverse direction and tied to evenly distribute stress at the coracoid process (Figures 6 and 7).

**FIGURE 4:**
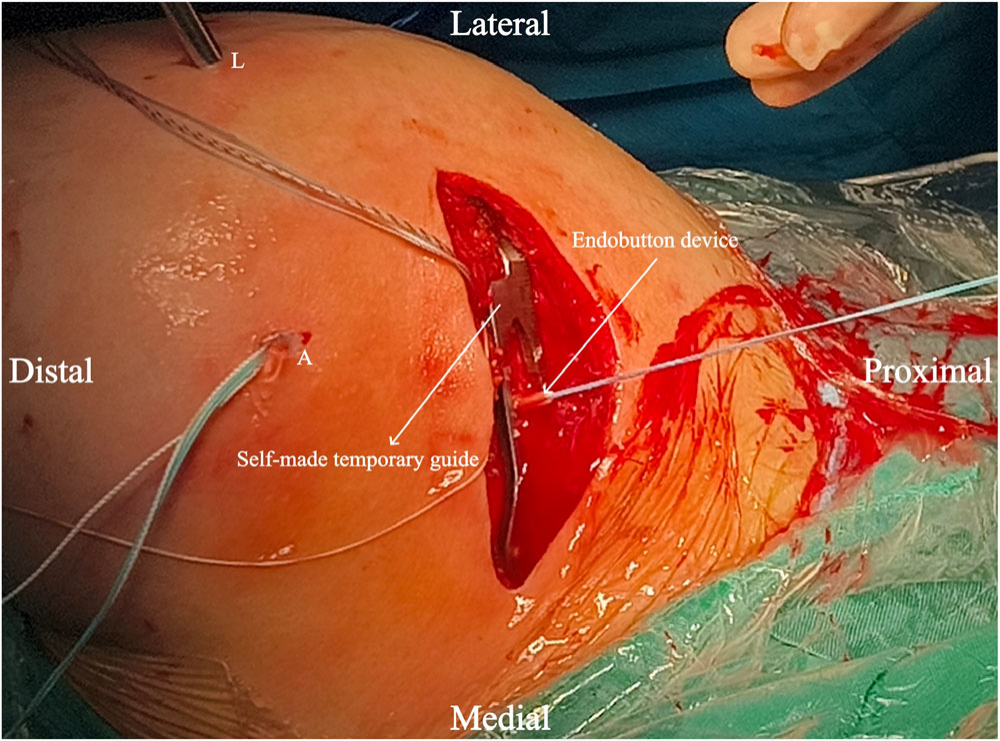
Insertion of the EndoButton device (right shoulder, left lateral position). (A, anterior portal; L, lateral portal.)

**FIGURE 5:**
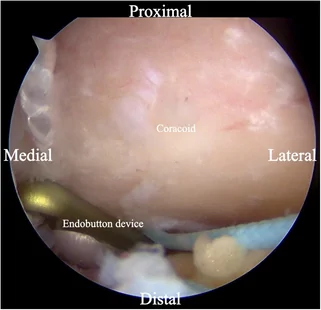
The EndoButton plate is positioned inferior to the coracoid process (right shoulder, left lateral position).

**FIGURE 6:**
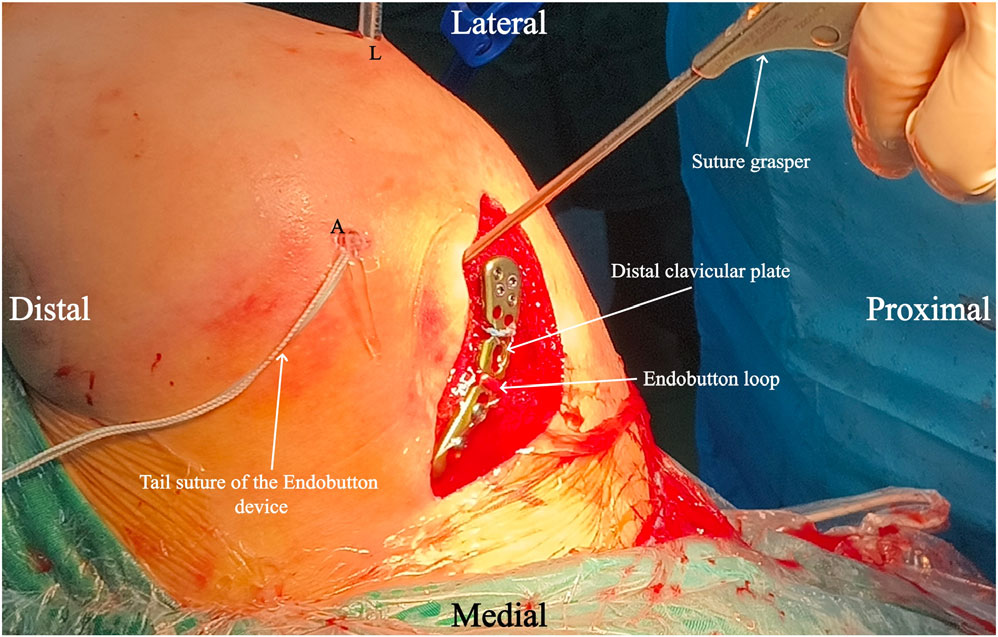
The coracoid sutures are retrieved in a reverse direction toward the superior surface of the clavicle, where they are tied to achieve even stress distribution at the coracoid process (right shoulder, left lateral position). (A, anterior portal; L, lateral portal.)

**FIGURE 7:**
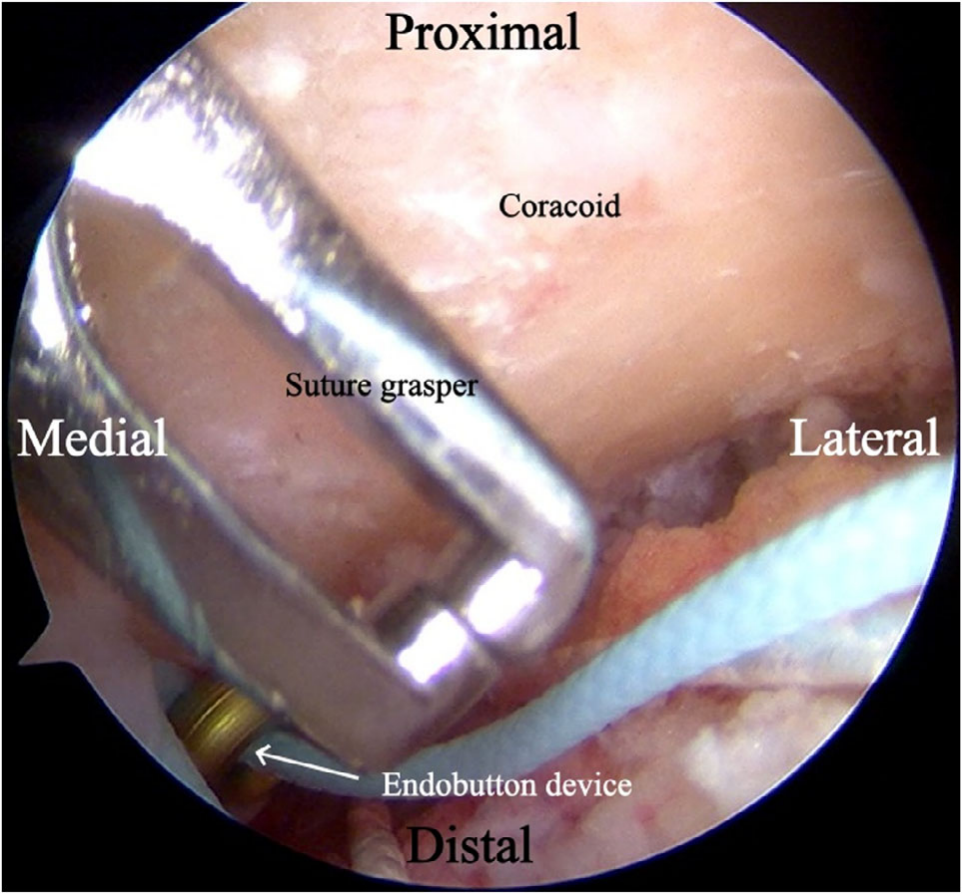
Reverse traction of the suture to distribute stress at the coracoid (right shoulder, left lateral position).

### Distal Clavicle Plate Fixation

Eventually, the temporary guide is removed, and a distal clavicle plate (Double Medical, Xiamen, Fujian, China) is positioned according to the fracture line. The plate is passed through the EndoButton loop, and fixation is achieved with screws placed at both the distal and proximal segments (Figure 8). The incision is then closed in a layer‐by‐layer fashion (Figure 9).

**FIGURE 8:**
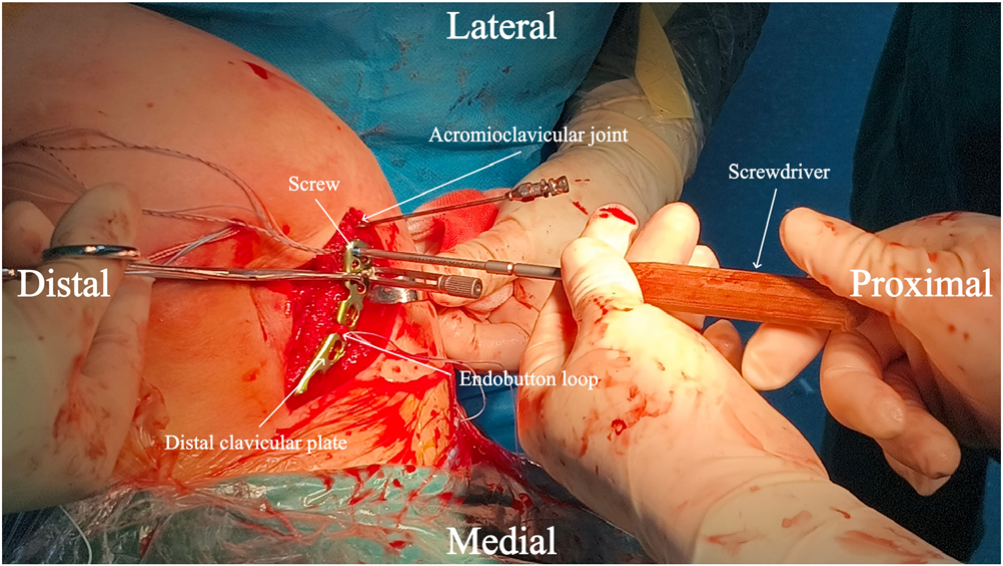
Plate fixation with screws (right shoulder, left lateral position).

**FIGURE 9:**
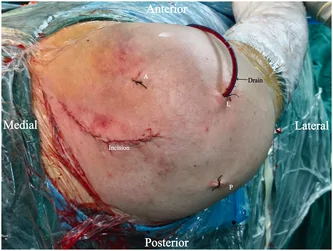
Layered closure of the incision (right shoulder, left lateral position). (A, anterior portal; L, lateral portal; P, posterior portal.)

### Postoperative Rehabilitation

Postoperatively, the affected limb is immobilized with a sling for 4 to 6 weeks. During the first 6 weeks, elbow flexion‐extension and pendulum exercises for the affected shoulder are permitted, with gradual passive range‐of‐motion exercises. Active range‐of‐motion exercises are initiated at 6 weeks postoperatively, followed by progressive resisted strengthening exercises at 8 weeks. The timing of return to athletic activities is individualized based on the patient's clinical and functional recovery.

## DISCUSSION

Herein, we describe a technique for treating Neer type IIB DCFs using a distal clavicle plate combined with an EndoButton device, enabling simultaneous fracture fixation and conoid ligament reconstruction with enhanced mechanical stability. In addition, the distal clavicle plate facilitates load sharing on the clavicle exerted by the EndoButton device, thereby lowering the risk of iatrogenic clavicle fractures.

Most previous studies have shown that combining a distal clavicle plate with coracoclavicular suspensory fixation provides superior biomechanical strength and improved clinical outcomes. In a cyclic compression test, this combined approach displayed a higher load‐bearing capacity compared with hook plate fixation.^6^ Besides, a comparative study by Yağar et al.^7^ found that using a distal clavicle plate combined with coracoclavicular fixation resulted in a higher bone union rate and better postoperative function than using a distal clavicle plate alone. Similarly, Wang et al.^8^ confirmed that coracoclavicular suspensory fixation led to a higher bone union rate and better functional recovery than hook plate fixation; although the former required a longer operative time, there was no significant difference in overall complication rates between the 2 methods.

Nevertheless, our modified technique has several limitations: (1) if implant‐related irritation warrants removal, the EndoButton device must be removed together with the plate. Removing the plate alone would compromise EndoButton fixation and may increase the risk of redislocation, particularly if the postoperative fixation period is short; (2) significant deviation of the coracoid tunnel could increase the risk of iatrogenic fracture; (3) the procedure has a steep learning curve, requiring advanced surgical experience (Tables 1 and 2).

**TABLE 1:** Pearls and Pitfalls of Surgery

**Pearls**	**Pitfalls**
1. Expose the base of the coracoid process	1. Position the bony tunnel as centrally as possible to reduce the risk of iatrogenic fracture
2. Achieve temporary fracture fixation with a Nice knot first	2. Select an EndoButton device with a size larger than the measured value to facilitate plate passage
3. If the coracoid tunnel is not centered, retrieve the traction suture in the reverse direction and knot it on the superior clavicular surface to distribute stress at the coracoid process	3. Avoid the use of large‐diameter drill bits
4. Pass the distal clavicle plate through the EndoButton loop to distribute stress at the clavicle	4. Manipulate the EndoButton device gently during insertion to prevent iatrogenic fracture

**TABLE 2:** Advantages and Disadvantages of Surgery

**Advantages**	**Disadvantages**
1. Allows concurrent management of other intra‐articular injuries or lesions	1. Relatively large incision (not minimally invasive), requiring further modification
2. Provides fracture fixation while restoring conoid ligament stability	2. In cases of implant‐related irritation, the plate and EndoButton device must be removed together
3. Distributes stress across both the coracoid and clavicular ends, reducing the risk of iatrogenic fractures	3. The technique has a steep learning curve and demands advanced surgical experience
4. Generally eliminates the need for a second surgery to remove the implant	

## DISCLOSURES

The authors (T.Z., C.H., H.F., X.H., Y.F., H.D., Y.Z.) declare that they have no known competing financial interests or personal relationships that could have appeared to influence the work reported in this article.
